# Protective Effect of Unacylated Ghrelin on Compression-Induced Skeletal Muscle Injury Mediated by SIRT1-Signaling

**DOI:** 10.3389/fphys.2017.00962

**Published:** 2017-11-24

**Authors:** Felix N. Ugwu, Angus P. Yu, Thomas K. Sin, Bjorn T. Tam, Christopher W. Lai, S. C. Wong, Parco M. Siu

**Affiliations:** ^1^Department of Health Technology and Informatics, Faculty of Health and Social Sciences, Hong Kong Polytechnic University, Kowloon, Hong Kong; ^2^School of Public Health, Li Ka Shing Faculty of Medicine, University of Hong Kong, Hong Kong, Hong Kong

**Keywords:** pressure sores, unacylated ghrelin, EX527, apoptosis, necroptosis, oxidative stress

## Abstract

Unacylated ghrelin, the predominant form of circulating ghrelin, protects myotubes from cell death, which is a known attribute of pressure ulcers. In this study, we investigated whether unacylated ghrelin protects skeletal muscle from pressure-induced deep tissue injury by abolishing necroptosis and apoptosis signaling and whether these effects were mediated by SIRT1 pathway. Fifteen adult Sprague Dawley rats were assigned to receive saline or unacylated ghrelin with or without EX527 (a SIRT1 inhibitor). Animals underwent two 6-h compression cycles with 100 mmHg static pressure applied over the mid-tibialis region of the right limb whereas the left uncompressed limb served as the intra-animal control. Muscle tissues underneath the compression region, and at the similar region of the opposite uncompressed limb, were collected for analysis. Unacylated ghrelin attenuated the compression-induced muscle pathohistological alterations including rounding contour of myofibers, extensive nucleus accumulation in the interstitial space, and increased interstitial space. Unacylated ghrelin abolished the increase in necroptosis proteins including RIP1 and RIP3 and attenuated the elevation of apoptotic proteins including p53, Bax, and AIF in the compressed muscle. Furthermore, unacylated ghrelin opposed the compression-induced phosphorylation and acetylation of p65 subunit of NF-kB. The anti-apoptotic effect of unacylated ghrelin was shown by a decrease in apoptotic DNA fragmentation and terminal dUTP nick-end labeling index in the compressed muscle. The protective effects of unacylated ghrelin vanished when co-treated with EX527. Our findings demonstrated that unacylated ghrelin protected skeletal muscle from compression-induced injury. The myoprotective effects of unacylated ghrelin on pressure-induced tissue injury were associated with SIRT1 signaling.

## Introduction

Pressure ulcers, also called bedsores or decubitus ulcers, are soft tissues injuries resulting from the application of prolonged pressure or friction to the body (Black et al., 2007). These ulcers are often identified and staged by the severity of symptoms, with stage I being the least and stage IV being the worst. The prevalence of pressure ulcers among ~6,000 patients surveyed in five European countries including Belgium, Italy, Portugal, United Kingdom, and Sweden has been reported to be ~18% (Vanderwee et al., 2007). The financial burden of pressure ulcers has been estimated to be over £2 billion in the United Kingdom (Bennett et al., 2004). In the United States, pressure ulcers have been associated with increased mortality, prolonged hospital stays, and hospital re-admission (Lyder et al., 2012). The association of pressure ulcer incidence and increased morbidity and mortality is substantiated by the observation that ~70% of 74 in-patients died within 6 months of developing pressure ulcers, with an average of 7 weeks from the ulcer onset to death (Brown, 2003). A recent report indicated that the mortality rate in patients with pressure ulcers was significantly higher than that in patients without pressure ulcers (Bauer et al., 2016). Furthermore, the staging of pressure ulcers positively relates to the pain severity of patients as well as the cost of treatment (Roth et al., 2004; Posthauer, 2012). Oot-Giromini et al. (1989) noted that the cost of preventing pressure ulcers was significantly less than the cost of treatment of pressure ulcers in the long term. Thus, early detection and prevention of pressure ulcers represent cost-effective remedies to alleviate the healthcare problem of pressure ulcers.

Ghrelin is a gastric peptide that exists in two main forms namely acylated ghrelin (AG) and unacylated ghrelin (UnAG). The receptor for AG has been identified as the growth hormone secretagogue receptor (GHSR) (Kojima et al., 1999). Although UnAG accounts for 80–90% of circulating ghrelin (Zhang et al., 2008), initial reports termed UnAG as an inactive peptide. This is mainly due to the inability of UnAG to activate GHSR at the physiologic level (Chen et al., 2009) and the lack of identified receptor or mediator that accounts for the activities of UnAG. Recently, some studies have begun to reveal the pro-survival role of UnAG, but in most instances the receptor or mediator responsible for the reported biological effects of UnAG has not yet been identified.

We have previously demonstrated the activation of apoptosis in the skeletal muscle of rats exposed to sustained, moderate compression. Our results indicated the increase of apoptotic markers including TUNEL index, apoptotic DNA fragmentation and cleavage of caspase 3, suggesting that apoptosis might play a role in the pathogenesis of deep tissue injury (Siu et al., 2009). We further confirmed our hypothesis via animal experiments showing that the administration of z-VAD-fmk, a caspase inhibitor, effectively abolished the elevation of apoptosis and the signs of muscle injury in the skeletal muscle of rats exposed to sustained, moderate compression (Teng et al., 2011). Besides apoptosis, necrosis has been implicated in the pathogenesis of pressure ulcers. For instance, Agrawal and Chauhan (2012) described pressure ulcer as “an area of unrelieved pressure usually over a bony prominence leading to ischemia, cell death, and tissue necrosis.” There is a dearth of studies investigating the role of necroptosis, a programmed form of necrosis, in pressure induced-deep tissue injury.

SIRT1 might be a potential signaling mediator involved in underpinning the cellular effects of UnAG. SIRT1 is a class III histone deacetylase, which regulates numerous cellular activities related to various physiological and pathologic signaling pathways (Wu et al., 2011; Lin et al., 2012). *In vitro* studies conducted by Shimada et al. (2014) have demonstrated that UnAG prevented microvascular endothelial cells from oxidative stress-induced apoptosis by stimulating the SIRT1 signaling pathway. Besides, UnAG treatment increased SIRT1 levels in endothelial cells of both ischemic and non-ischemic muscles in ob/ob obese mice (Togliatto et al., 2015). *In vitro* studies have also revealed that UnAG protected muscle cells. UnAG has been demonstrated to prevent dexamethasone-induced atrophy in C2C12-derived myotubes via mammalian target of rapamycin complex 2 (mTORC2) pathway (Porporato et al., 2013). Sheriff et al. (2012) have also demonstrated that protein catabolism induced by combined treatment of two pro-inflammatory factors, tumor necrosis factor alpha (TNFα) and interferon gamma (IFNγ) in cultured C2C12 myotubes, was abolished by UnAG administration via PI3K signaling pathway. Collectively, there is a dearth of knowledge on the biological effects of UnAG on skeletal muscle. The present study tested the hypothesis that UnAG would protect skeletal muscle from pressure-induced injury and the myoprotective effects of UnAG are mediated through SIRT1 signaling pathway.

## Materials and methods

### Animals

Sprague Dawley rats (*N* = 15) weighing between 280 and 300 g were housed in a light, temperature, and humidity controlled environment under standard conditions, with a 12-h light-dark cycle and free access to water and feed. Water and bedding consisting of sawdust were changed weekly, whereas the animal room was cleaned daily. Animal husbandry and compression protocol were conducted in full accordance with the approval of the Animal Subjects Ethics Subcommittee of The Hong Kong Polytechnic University (ASESC Case #: 14-15/23-HTI-R-OTHERS).

### Compression injury protocol

The induction of pressure ulcer in Sprague Dawley rats was performed in accordance with our established protocol (Sin et al., 2013). Briefly, rats were anesthetized with a ketamine and xylaxine mixture via intraperitoneal injection. The hairs on both hind limbs were gently shaved while animals were under anesthesia. Animals were subjected to a 2-day compression protocol. On the first day of compression, a static pressure of 100 mmHg was applied to the tibialis region of the right hind limb for 6 h via a compression indentor. A laser Doppler flow meter (DRT4, Moor Instruments, Axminster, UK) with a contact probe (DP1T/7-V2) was used to monitor the blood flow of the compression site. Anesthesia was ensured throughout the compression by intraperitoneal injection of one-third of the initial anesthetic mixture delivered when needed. The left uncompressed hind limb served as intra-animal control. After an 18-h rest, the entire compression protocol was repeated on the second day. Animals were sacrificed 18-h after the second compression via CO_2_ ventilated euthanasia. Muscle tissue underneath the compression region and muscle tissue at a similar region of the opposite uncompressed limb were harvested and frozen in liquid nitrogen, and then stored at −80°C for further analyses.

### Treatment of UnAG and EX527

Animals were randomly assigned into three groups (*n* = 5 per group) and received intraperitoneally one of the following treatments for 2 days: saline, UnAG (100 μg/kg, injected before and after compression) or UnAG (100 μg/kg, injected before and after compression) co-treated with EX527 (1 mg/kg injected before compression). EX527 is a potent pharmacological inhibitor of SIRT1. The dose of UnAG adopted in the present study was reported in previous studies (Nagaya et al., 2001; Li et al., 2006). The dose and route of administration of EX527 was adopted from a previous study (Kemelo et al., 2014). UnAG and EX527 were purchased from Tocris Bioscience (Catalog numbers 2,951 and 2,780, respectively).

### Histological analysis

Muscle tissues were separated into two halves transversely. Twelve micrometers-thick frozen muscle tissue cross-sections were prepared in a cryostat at −20°C. Tissue sections were air-dried at room temperature for 30 min and fixed with 10% formalin at room temperature for 10 min before being stained in Mayer's Hematoxylin and 1% Eosin in CaCl_2_. Thereafter, sections were dehydrated, mounted and examined under a confocal microscope. Interstitial nuclei and muscle nuclei were counted and an area of interstitial space was quantified by using ImageJ software (National Institutes of Health, USA). The number of nuclei within myofibers was normalized to the number of myofibers in the field. All the histological data were presented as averages from four random, non-overlapping image fields captured under a 20x objective.

### Tunel and dystrophin staining analysis

Apoptotic nuclei were detected using double immunofluorescence terminal dUTP nick-end labeling (TUNEL *in situ* Cell Death Detection Kit, Roche Applied Science) and dystrophin staining, according to the manufacturer's instructions. Briefly, 20-μm thick frozen muscle cross-sections were air dried, fixed in 4% paraformaldehyde in phosphate buffered saline, pH 7.4, at room temperature for 20 min, permeabilized with 0.2% Triton X-100 in 0.1% sodium citrate at 4°C for 2 min, and incubated in TUNEL reaction mixture in a humidified chamber at 37°C for 1 h in the dark. Negative control experiments were performed by omission of terminal deoxynucleotidyl transferase enzyme in the TUNEL reaction mixture. Tissue sections were then labeled with dystrophin to visualize the sarcolemmal membrane by incubating with anti-dystrophin mouse monoclonal antibody (D8168, Sigma-Aldrich, 1: 400 dilution) followed by Cy3-conjugated anti-mouse IgG (C2181, Sigma-Aldrich, 1:400). The sections were then mounted with 4', 6-diamidino-2-phenylindole (DAPI, H-1200, Vector Laboratories) to visualize the nuclei. DAPI- and TUNEL-stained nuclei and dystrophin staining were examined under a confocal microscope (Confocal Microscope C1, Nikon). Images of five random and non-overlapping fields were examined at an objective magnification of 20 × for analysis. Data were expressed as TUNEL apoptotic index calculated as the number of muscle-related TUNEL-positive nuclei (nuclei that were within muscle cells or on dystrophin staining) divided by the number of total nuclei multiplied by 100.

### Immunofluorescence staining of bax and dystrophin

Twenty-micrometer-thick frozen sections were air-dried at room temperature and fixed in cold acetone for 10 min. Sections were then rehydrated with phosphate-buffered saline (PBS) for 10 min. Background activity was minimized by blocking the sections with 1% BSA in PBST for 30 min at room temperature. Sections were then incubated with rabbit IgG anti-bax (493, Santa Cruz 1:50) and mouse IgG anti-dystrophin (D8168, Sigma-Aldrich, 1: 500) in 1% BSA in PBST in a humidified chamber overnight at 4°C. Negative controls were performed by eliminating the primary antibody. Sections were washed three times with PBS with 5 min intervals between each and incubated with fluorescein-conjugated anti-rabbit IgG (Alexa Fluor 488, Life Technologies, 1:300) and Cy3-conjugated anti-mouse IgG (C2181, Sigma-Aldrich, 1:400) for 1 h. Sections were then washed with PBS and mounted with mounting medium containing DAPI. The sections were finally examined under a confocal microscope (Confocal Microscope C1, Nikon) with microscopy imaging software (SPOT advanced software, Diagnostic Instruments). Images were taken using the objective of x20. Bax signal was quantified using ImageJ by following a procedure reported previously (McCloy et al., 2014). Briefly, an outline was drawn around each muscle cell for selecting the cell as region of interest (ROI). The integrated density of each ROI was measured, along with several adjacent background readings. The total corrected cellular fluorescence (TCCF) was calculated as integrated density—(area of selected cell × mean fluorescence of background readings).

### Subcellular protein fractionation and western blot

Proteins were extracted as previously described (Sin et al., 2013). The protein contents were quantified using Bradford Assay. The abundance of interested protein was determined by Western blotting. Briefly, the protein samples were boiled at 95°C in Laemmeli buffer with 5% β-mercaptoethanol, fractioned by SDS-PAGE. They were then transferred onto a PVDF membrane, blocked with 5% milk for an hour, and probed with one of the following primary antibodies at 4°C overnight: anti-SIRT1 (15404, Santa Cruz, Santa Cruz, CA, USA), anti-RIP1 (7881, Santa Cruz), anti-RIP3 (135170, Santa Cruz), anti-NOS2 (650, Santa Cruz), anti-p53 (56179, Santa Cruz), anti-phospho-p53 (101762, Santa Cruz), anti-Bax (493, Santa Cruz), anti-AIF (13116, Santa Cruz), NF-kB p65 (C22B4, Cell Signaling), anti-phospho-p65 NF-kB (Ser536) (3031, Cell signaling), or anti-acetyl-p65 NF-kB (K310) (19870, Abcam). The membranes were washed three times at an interval of 15 min each, probed with the appropriate secondary antibodies, either anti-mouse IgG or anti-rabbit IgG horseradish peroxidase (HRP)-conjugated antibodies (1:4,000; Cell Signaling, Beverly, MA, USA) for 1 h, followed by three washes and the application of luminal reagent (NEL103001EA, Perkin Elmer, Waltham, MA, USA). The signals were captured via BioRad Chemidoc System. Beta-tubulin was used as the internal control and for normalization.

### Apoptotic cell death enzyme-linked immunosorbent (ELISA) assay

The Cell Death Detection ELISA kit (Roche Diagnostics, Indianapolis, IN, USA) was employed to determine the apoptotic DNA fragmentation according to the instructions of the manufacturer. In this procedure, the wells of a microplate were coated with primary mouse monoclonal histone antibody in coating solution at 4°C overnight. Thereafter, samples were incubated in coated wells at room temperature for 90 min, followed by washes and incubation with conjugated solution containing secondary peroxidase-conjugated anti-DNA-POD mouse monoclonal antibody. Reaction in the absence of conjugation solution served as the negative control. After washes, 2,2'-azino-di-(3-ethyl-benzthiazoline sulphonate) substrate was added to the microplate to determine the retained amount of peroxidase colorimetrically by measuring the absorbance at a wavelength of 405 nm via a spectrophotometer. The optical density (OD) was normalized to the milligrams of protein used in the assay and presented as apoptotic DNA fragmentation index.

### SIRT1 deacetylation assay

Deacetylase activity of SIRT1 was assessed by a fluorometric assay (Cyclex, Nagoya, Aichi, Japan) in accordance with the manufacturer's instructions. Briefly, a reaction mixture containing 1 mM fluoro-substrate peptide, 5 mAU lysylendopeptidase, 2 mM NAD^+^, 50 mM Tris-HCl, 0.5 mM DTT-containing SIRT1 assay buffer, 1 μm Trichostatin A (a NAD^+^-independent histone deacetylase inhibitor) was prepared. The reaction was initiated by adding 5 μl protease inhibitor-free muscle protein extracts under thorough mixing. Fluorescence intensity (excitation 340 nm, emission 460 nm) was measured by using a microplate fluorometer (Infinite F200, Tecan, Mannedorf, Switzerland). All readings were normalized to 1 mg of protein content of the respective samples.

### Statistical analyses

Statistical analyses were performed using the SPSS 22.0 software package (IBM, Chicago, IL, USA). In all experimental studies, the contralateral limb served as the intra-animal control. A normality test was performed to examine data distribution. Normally distributed data were compared using one-way analysis of variance (ANOVA) followed by Tukey's Honestly Significant Difference (HSD) *post-hoc* test. Non-normal data were analyzed by Kruskal-Wallis H test followed by Dunn-Bonferroni *post-hoc* test. All data were expressed as mean ± standard error of the mean (SEM). Significance level was set at p < 0.05.

## Results

### Unacylated ghrelin attenuated histopathological alterations of skeletal muscle after compression

**Figure 1:**
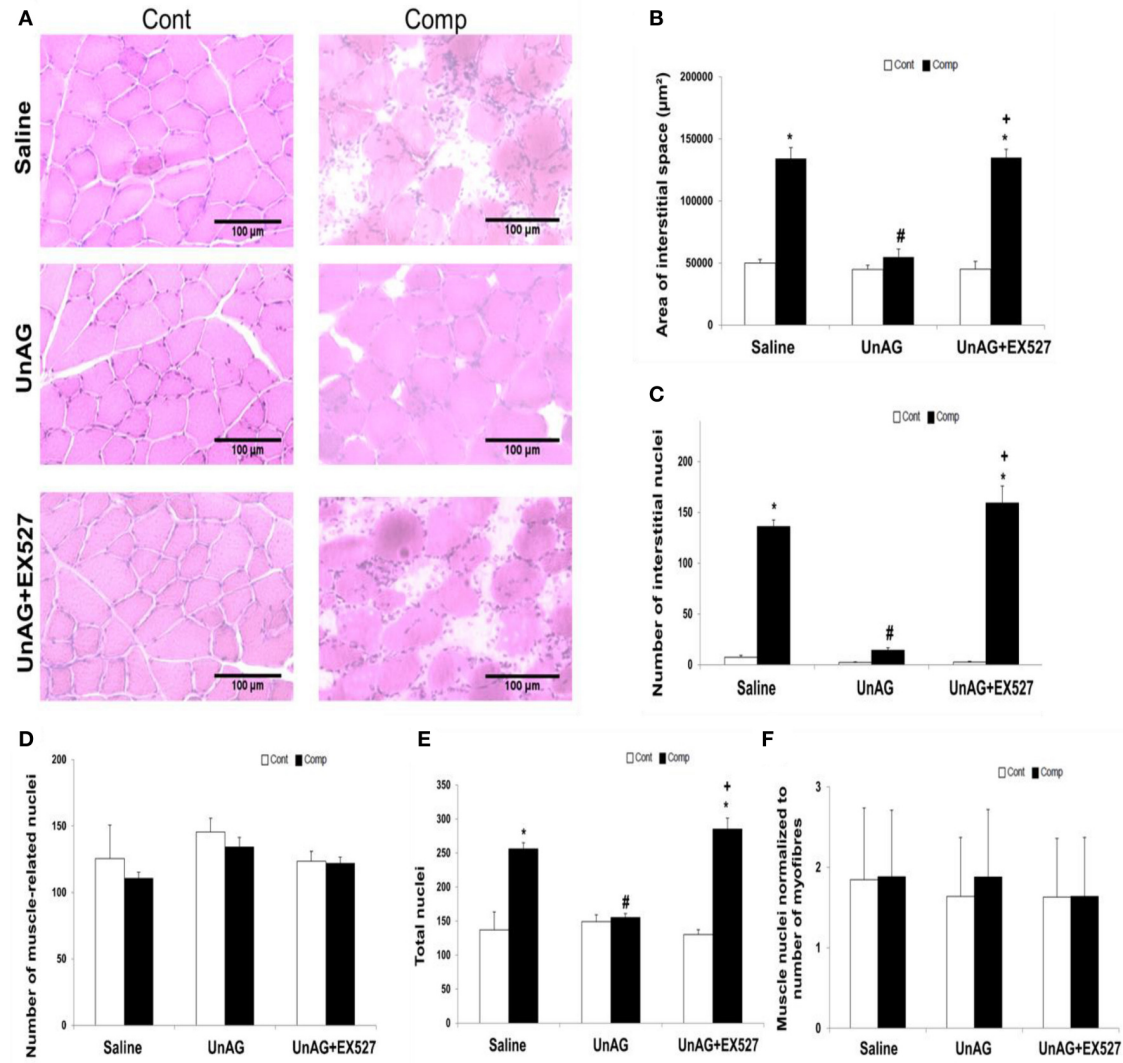
Histological analyses. UnAG abolished abnormal muscle histology induced by moderate compression but this effect was vanished when co-treated with EX527 **(A)**. The elevation of area of interstitial space induced by compression injury was alleviated by UnAG **(B)**. The reduction of the number of interstitial nuclei induced by UnAG in the compressed muscle was mitigated by the co-treatment of EX527 **(C)**. No compression or treatment effect was observed for the number of muscle-related nuclei **(D)**. The total number of nuclei was significantly different between compressed and uncompressed muscles in all groups except in the group treated only with UnAG **(E)**. Neither compression nor drug treatment affected the number of muscle nuclei normalized to the number of myofibers **(F)**. ^*^p < 0.05, compressed muscle compared to uncompressed control muscle; #*p* < 0.05, UnAG compared to Saline; ^+^*p* < 0.05, UnAG+EX527 compared to UnAG; Cont, control muscle; Comp, compressed muscle.

The effects of compression injury and drug treatment on muscle histology are shown in the representative pictures (Figure 1A). The area of interstitial space in compressed muscle was increased 2.7-fold when compared to uncompressed muscle in saline group (Figure 1B). UnAG, but not in combination with EX527, prevented the elevation of the area of interstitial space in the compressed muscle (Figure 1B). Our histological results revealed that the number of interstitial nuclei was 18.9-fold higher in the compressed muscle relative to the uncompressed muscle in saline group (Figure 1C). Increase in the number of interstitial nuclei was attenuated by UnAG (Figure 1C). The number of muscle-related nuclei was not significantly different between compressed and uncompressed muscles in all groups (Figure 1D). The total number of nuclei was significantly different between compressed and uncompressed muscles in all groups except the group treated only with UnAG. The total number of nuclei in compressed muscle was increased 1.9-fold when compared to the uncompressed muscle in saline group (Figure 1E). Furthermore, the number of muscle nuclei normalized to the number of myofibers remained unchanged after compression in all groups (Figure 1F).

### Unacylated ghrelin opposed increase in TUNEL index

**Figure 2:**
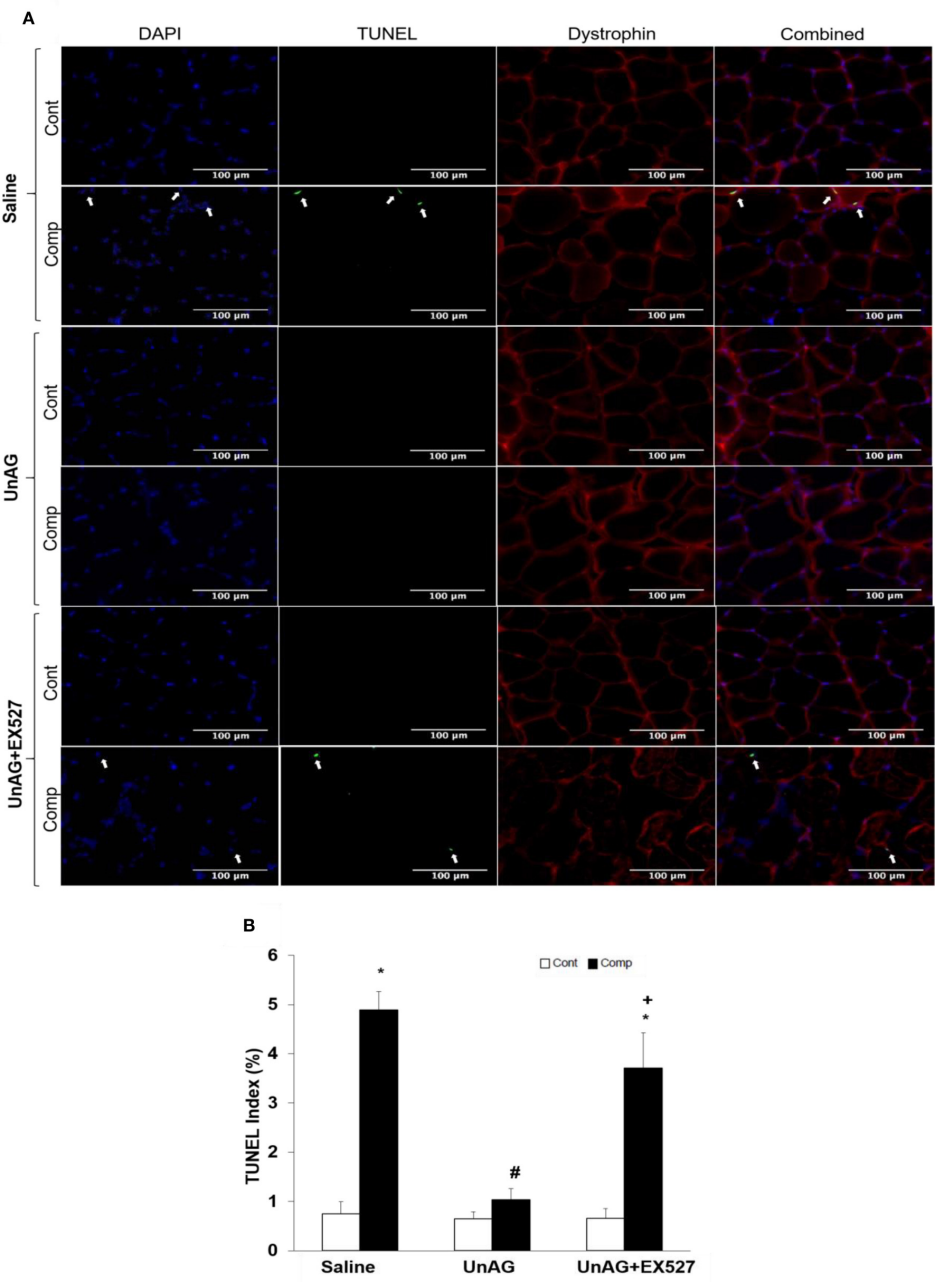
TUNEL and dystrophin staining. Apoptotic nuclear DNA breaks in muscle tissue were measured using the technique of TUNEL staining **(A)** and were expressed as TUNEL index **(B)**. Representative images of TUNEL and dystrophin staining of compressed and uncompressed muscles **(A)**. Immunofluorescence labeling of dystrophin (red) was performed to identify the localization of the TUNEL-positive nuclei (green) with regard to muscle sarcolemmal membrane and nuclei were labeled with DAPI (blue) **(A)**. UnAG opposed the increase of TUNEL positive nuclei induced by moderate compression but this protective effect was absent when co-treated with EX527 **(A)**. TUNEL index was higher in compressed muscles of saline and UnAG+EX527 groups compared to compressed muscle of UnAG group **(B)**. Arrows indicate TUNEL-positive nuclei. ^*^p < 0.05, compressed muscle compared to uncompressed control muscle; #*p* < 0.05, UnAG compared to Saline; ^+^*p* < 0.05, UnAG+EX527 compared to UnAG; Cont, control muscle; Comp, compressed muscle; DAPI, 4′,6-diamidino-2-phenyl-lindole.

Immunofluorescence staining of sarcolemmal dystrophin was performed together with TUNEL staining aimed to facilitate the identification of muscle-associated nuclei (Figure 2A). Our TUNEL/dystrophin staining analysis indicated that the percentage of TUNEL-positive apoptotic muscle-related nuclei increased 6.6-fold in the compressed muscle compared to uncompressed muscle in saline group. Increase in the TUNEL index was attenuated by UnAG (Figure 2B). The percentage of TUNEL-positive nuclei was 5.7-fold greater in compressed muscle treated with UnAG+EX527 compared with contralateral uncompressed tissue (Figure 2B).

### Unacylated ghrelin decreased abundance of muscle bax

**Figure 3:**
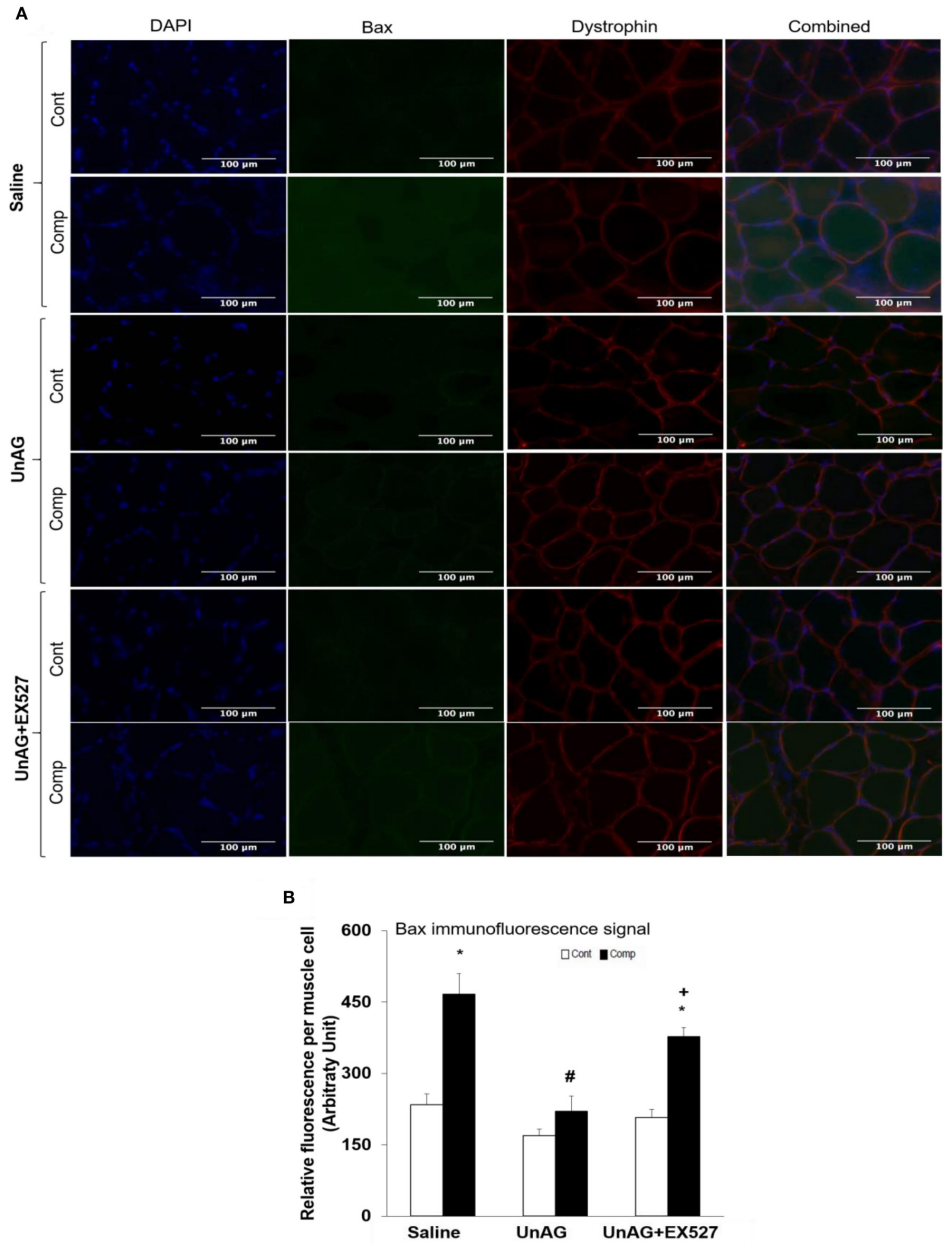
Immunofluorescence staining of Bax and dystrophin. Representative images of compressed and uncompressed muscles labeled with anti-dystrophin (red) and anti-bax (green) **(A)**. Muscle Bax signal was increased in compressed muscles treated with saline and UnAG+EX527 **(A)**. In contrast, muscle Bax abundance was decreased in compressed muscles treated with only UnAG **(A)**. Quantification of relative fluorescence intensity of Bax **(B)**. ^*^p < 0.05, compressed muscle compared to uncompressed control muscle; #*p* < 0.05, UnAG compared to Saline; ^+^*p* < 0.05, UnAG+EX527 compared to UnAG; Cont, control muscle; Comp, compressed muscle.

Immunofluorescence staining of Bax (green) and dystrophin (red) were also performed to determine the expression of Bax in both compressed and uncompressed muscles (Figure 3A). Increased Bax expression was observed in compressed muscles of saline and UnAG+EX527 groups but not in compressed muscle of UnAG group (Figure 3A). Quantification of relative fluorescence intensity revealed that muscle Bax signal was increased by 2-fold in the compressed muscle compared to uncompressed muscle in saline group while muscle Bax signal was increased by 1.8-fold in the compressed muscle compared to uncompressed muscle in UnAG+EX527 group (Figure 3B).

### Unacylated ghrelin abolished the increase in necroptosis proteins, exerted anti-apoptotic effects, and preserved SIRT1 enzymatic activity in compressed muscle

**Figure 4:**
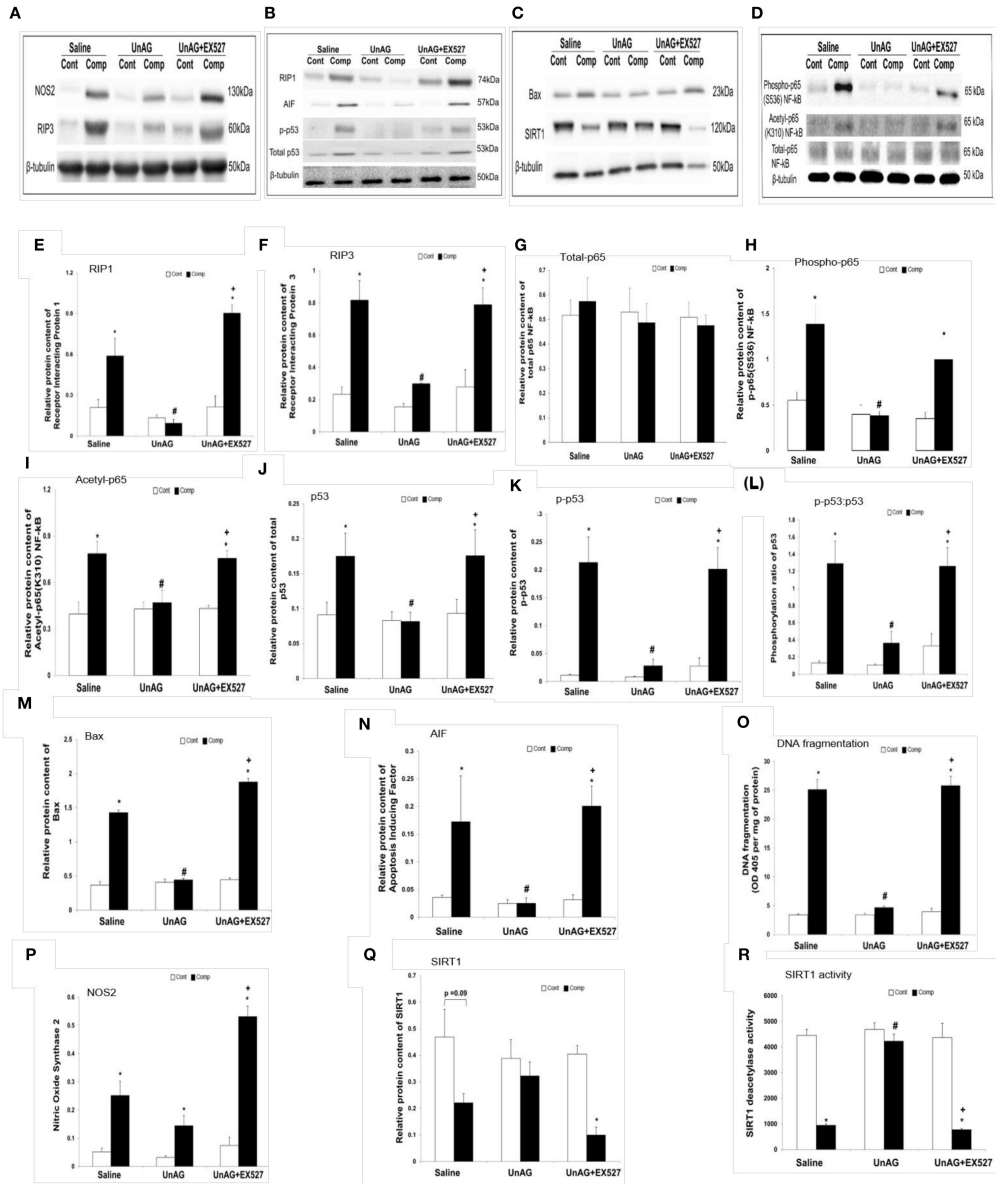
Immunoblot and biochemical analyses. For the immunoblotting, all samples were probed for the examined protein with the respective antibody on the same membrane which underwent the same probing and washing procedure. The representative blot pictures are shown in **(A–D)**. UnAG, but not in conjunction with EX527, blunted the increase in protein abundance of RIP1 in compressed muscle **(E)**. Similar pattern was observed in the protein abundance of RIP3 **(F)**. Total p65 NF-kB protein abundance did not change in the compressed muscle when compared to uncompressed muscle in all groups **(G)**. The immunoblot analyses revealed the suppressive effects of UnAG, but not in conjunction with EX527, on the abundances of phospho-p65 NF-kB **(H)**, acetyl-p65 NF-kB **(I)**, p53 **(J)**, phospho-p53 **(K)**, phosphorylation ratio of p53 **(L)**, Bax **(M)**, and AIF **(N)**. The anti-apoptotic effect of UnAG was confirmed by apoptotic DNA fragmentation index **(O)**. The protein content of NOS2 was significantly elevated in the compressed muscles in all groups, irrespective of drug administered **(P)**. SIRT1 protein abundance was tended to decrease in the compressed muscle when compared to uncompressed muscle in saline group **(Q)**. SIRT1 protein abundance was significantly decreased in muscle co-treated with UnAG and EX527 after compression **(Q)**. The deacetylase activity of SIRT1 was significantly decreased after compression in all groups except in compressed muscle treated only with UnAG **(R)**. ^*^p < 0.05, compressed muscle compared to uncompressed control muscle; #*p* < 0.05, UnAG compared to Saline; ^+^*p* < 0.05, UnAG+EX527 compared to UnAG; Cont, control muscle; Comp, compressed muscle.

Representative blots of examined proteins are shown in Figures 4A–D. To investigate the necroptosis signaling, RIP1 and RIP3 protein abundances were examined. In muscle of saline group, RIP1 was significantly increased 1.8-fold in response to compression (Figure 4E). This compression-induced elevation of RIP1 was abolished by UnAG treatment but not in conjunction with EX527 (Figure 4E). Similarly, RIP3 significantly increased 2.5-fold in the compressed muscle relative to uncompressed muscle in the saline group (Figure 4F). The elevation of RIP3 was attenuated by UnAG treatment but not in combination with EX527 (Figure 4F). There was no significant difference in total p65 NF-kB protein between the compressed and uncompressed muscles in all groups (Figure 4G). In saline group, the protein abundances of phospho-p65 NF-kB, acetyl-p65 NF-kB, total p53, phospho-p53, Bax, AIF, and phosphorylation ratio of p53 were increased in the compressed muscle by 2.5, 2, 1.9, 18.7, 3.9, 4.8, and 9.8-fold, respectively, relative to the uncompressed muscle (Figures 4H–N). These increases were all attenuated by UnAG treatment but not in combination with EX527 (Figures 4H–N). Similarly, apoptotic DNA fragmentation index was elevated 7.4-fold in the compressed muscle when compared to the uncompressed muscle in saline group (Figure 4O). UnAG treatment, but not in combination with EX527, inhibited the increase in apoptotic DNA fragmentation index as induced by compression (Figure 4O). The protein content of NOS2 was significantly elevated in the compressed muscles relative to the uncompressed muscles in all groups (Figure 4P). The protein content of NOS2 was significantly elevated by 3.8, 3.6, and 6.1-fold in the compressed muscles relative to the uncompressed control muscles in saline, UnAG, and UnAG+EX527 groups, respectively, (Figure 4P). SIRT1 protein abundance tended to decrease in compressed muscles when compared to the uncompressed muscles in saline group (*p* = 0.09) (Figure 4Q). The protein abundance of SIRT1 significantly decreased in the compressed muscle relative to the uncompressed muscle in UnAG+EX527 group. In saline group, the deacetylase activity of SIRT1 significantly decreased by 72% in the compressed muscle relative to the uncompressed muscle (Figure 4R). This compression-induced decrease in SIRT1 deacetylase activity was not found in the compressed muscle treated with UnAG (Figure 4R).

## Discussion

The present study demonstrated the protective effect of UnAG on muscle compression injury as shown by the maintenance of muscle histology after prolonged moderate compression. Our results illustrated that the protective effect of UnAG on muscle compression could be attributed to favorable alterations of necroptosis and apoptosis signaling in skeletal muscle, which probably involved the mediation of SIRT1 signaling pathway.

Studies from our laboratory have demonstrated that UnAG prevented doxorubicin-induced myocardial apoptosis and fibrosis (Pei et al., 2014), exerted protective effect on diabetic cardiomyopathy (Pei et al., 2015) and inhibited doxorubicin-induced apoptosis in skeletal muscle (Yu et al., 2014). A recent investigation has revealed that 4-day administration of UnAG in lean rats inhibited the production of inflammatory cytokines and mitochondrial reactive oxygen species in gastrocnemius muscle (Cappellari et al., 2016). Notably, the specific molecule or receptor that mediates the reported protective effects of UnAG has not yet been recognized. SIRT1 is known to regulate pro-survival events and play roles in lifespan extension, apoptosis, age-related disorders, inflammation, and cancers (Wu et al., 2011; Lin et al., 2012). Here, we reported a possible role of SIRT1 in mediating the myoprotective effects of UnAG on compression-induced muscle injury.

Necrosis is a complex form of caspase-independent cell death whereas necroptosis is the term used to describe necrosis that is dependent on necroptosis proteins RIP1 and RIP3 (Vanlangenakker et al., 2012). Different factors including oxidative stress, chemical toxins, and DNA damage are known to induce necrotic cell death (Vanlangenakker et al., 2012) and the examination of RIP1 and RIP3 serves as a marker for determining necroptosis (Zhang et al., 2009, 2011). In this study, the observed activation of necroptosis might relate to ischemia-reperfusion injury in accordance with the findings reported by Fiorillo et al. (2006) that hypoxia-reoxygenation induced necrosis-dependent activation of poly(ADP-ribose) polymerase 1 (PARP1). According to our observation that UnAG downregulated both necroptosis proteins in the absence of the inhibitor for SIRT1, we interpreted that UnAG might have maintained the cyto-architecture of the compressed muscle by attenuating necroptosis, which is a cellular destructive process. Our data indicated that the interaction of UnAG with SIRT1 might play a role in coordinating necroptosis during pressure-induced tissue injury, suggesting that SIRT1 might be able to serve as the potential molecular target for further unraveling the pathogenesis of pressure ulcers as well as the myoprotective effect of UnAG in pressure ulcers. Chen et al. (2017) revealed recently that NADPH oxidase 4 (NOX4) increased the expression of RIPK1 and simultaneously activated nuclear factor kappa B (NF-kB) via increased phosphorylation of p65 subunit of NF-kB, in the heart tissue of transverse aortic constriction rats. Notably, gene silencing of NOX4 by specific siRNA effectively opposed the upregulation of RIPK1 and phosphorylation of p65 subunit of NF-kB, suggesting that NOX4 might be required for the activation of necroptosis and NF-kB signaling pathways (Chen et al., 2017). Resveratrol, the potent SIRT1 agonist, suppressed NOX4 upregulation in the muscle of dystrophin-deficient mice, whereas SIRT1 knockdown in cultured C2C12 myoblasts resulted in NOX4 protein upregulation (Hori et al., 2011). Although the mechanism by which SIRT1 negatively regulates NOX4 expression remains elusive (Kuno and Horio, 2016), our data suggested that UnAG might have preserved SIRT1 activity, leading to repression of NOX4 function and concomitant inhibition of RIP1, RIP3, phospho-p65, and acetyl-p65 expressions.

The phosphorylation of RIP1 is considered a hallmark of necroptosis. Using kinase assay, multiple RIP1 phosphorylation sites, including Ser14/15, Ser20, Ser161, and Ser166 were discovered, suggesting that phosphorylation might regulate RIP1 kinase activity (Degterev et al., 2008). In addition, *in vitro* autophosphorylation assay showed that S161A-RIP1 mutant was significantly less active compared with the wild-type kinase, implying Ser161 autophosphorylation site of RIP1 is critical for the catalytic regulation of RIP1 activity (Degterev et al., 2008). In a separate investigation, McQuade et al. (2013) tested the function of Ser161 but reported that alanine substitution only tended to decrease RIP1 kinase activity in HEK293T cells. Intriguingly, S161A-RIP1 increased TNF-induced apoptosis and restored necroptosis to similar levels achieved by wild-type RIP1 in Jurkat cells (McQuade et al., 2013). Their results demonstrated the potency of S161A-RIP1 in mediating necroptosis, despite minor reduction in kinase activity. While our study focused primarily on the expression levels of RIP1 and RIP3, it provides a framework for further analyses of the molecular mechanisms of RIP1 and RIP3 kinase activation, with emphasis on their phosphorylation residues in pressure-induced deep tissue injury.

Reactive oxygen species and antioxidants are opposing forces that need to be appropriately balanced (Pipinos et al., 2008). NOS2 is an enzyme that promotes the formation of large quantities of nitric oxide, a free radical with a high affinity for other free radicals (Ponnuswamy et al., 2009). For instance, the interaction of nitric oxide with superoxide produces peroxynitrite, which causes protein nitration and DNA damage (Beckman et al., 1990). Studies a decade ago indicated that the peripheral administration of AG attenuated NOS2 protein expression, which was upregulated by gastric ischemic injury in rats and decreased *in vivo* reactive oxygen species generated by human polymorphoneuclear cells (El Eter et al., 2007). In addition, AG enhances the expression of key antioxidant enzymes including zinc and manganese superoxide dismutases, catalase, glutathione peroxidase, and glutathione reductase but reduces expression of NOS2 in liver (Dobutovic et al., 2014). Although the beneficial effects of AG on the antioxidant enzymes, as well as NOS2, were examined in the absence of any pathophysiological condition, previous findings have suggested that AG might regulate the oxidative stress enzymes and NOS2 via Akt/ERK1/2 pathway. It remains to be proven whether administration of AG or UnAG may confer antioxidative protection in skeletal muscles subjected to compression-induced injury. Studies from our laboratory revealed that the transcript content of NOS2 was elevated in skeletal muscle after compression (Sin et al., 2013). Recently, UnAG was found to preserve gastrocnemius muscle mass in male Wistar rats with chronic kidney disease (as induced via nephrectomy) by normalizing the mitochondrial reactive oxygen species production, inflammatory mediators, and insulin signaling (Cappellari et al., 2015). Nonetheless, our results showed that UnAG treatment did not reverse the increased abundance of NOS2 in the compression-injured skeletal muscle. Instead, our findings suggested a role of SIRT1 in mediating the effect of UnAG on NOS2 expression as, simultaneously, UnAG tended to decrease NOS2 but increased SIRT1 activity in the absence of EX527. *In vitro* study has revealed that SIRT1 deletion elevated the expressions of two hypoxia-responsive genes, interleukin 1β and NOS2 (Takikawa et al., 2016). Hypoxia is a fundamental pathology for ischemic-reperfusion diseases including pressure ulcers (Zhao et al., 2000). Given that UnAG tended to reduce NOS2 expression, possibly by interacting with SIRT1, our data suggested that at higher doses, UnAG might exert a beneficial effect by diminishing hypoxia-related pathologies associated with compression-induced muscle injury.

It is difficult to evaluate the exact contribution of SIRT1 expression toward its *in vivo* activity (Revollo and Li, 2013). However, it has been proposed that complementary system exists whereby conditions that impact SIRT1 activity simultaneously shift with SIRT1 expression level. For instance, SIRT1 activity and expression are known to be increased by higher levels of NAD+ generated in low-energy states, (Revollo and Li, 2013). Our laboratory has previously reported that oxidative stress damage occurred in compression-induced deep tissue injury (Sin et al., 2013). Since compression injury represents a classic example of low-energy condition, the current findings suggested that oxidative stress associated with compression injury might have decreased SIRT1 activity, leading to a concomitant decrease in SIRT1 expression level.

NF-kB is a family of inducible transcription factors that regulates vital processes including inflammation, apoptosis, cell proliferation, and differentiation (Rothgiesser et al., 2010). NF-kB activation induces muscle wasting via increased transcription of muscle-specific atrogenes including muscle ring-finger 1 (Murf1) and atrogin-1 (MAFbx) (Cai et al., 2004; Judge et al., 2007). Specifically, p65 subunit of NF-kB and p53 are key transcription factors in the modulation of inflammation and apoptosis, respectively (Nakazawa et al., 2017). SIRT1 deacetylates p65 NF-kB and p53, thereby reducing inflammation and apoptosis (Vaziri et al., 2001; Yang et al., 2012). SIRT1 inhibits NF-kB transcription by direct deacetylation of p65 at lysine 310 (Yeung et al., 2004), which attenuates cardiac oxidative stress in rats (Bagul et al., 2015). A recent study reported that NOS2-mediated inactivation of SIRT1 increased both acetylation and activity of p65 NF-kB and p53 (Shinozaki et al., 2014). Interestingly, our results indicate that compression-induced apoptosis in muscle might be enhanced by an increase in p53 and NF-kB activities. Our results further revealed that UnAG increased SIRT1 activity but inhibited both phosphorylation and acetylation of p65, total and phospho-p53 induced by moderate compression. It is noteworthy that these alterations were diminished by co-administration with EX527, suggesting that the anti-apoptotic effects of UnAG might be SIRT1-dependent.

Previously, we reported the activation of apoptosis in conjunction with pathohistological damage in skeletal muscle in response to moderate compression (Siu et al., 2009). Additionally, we demonstrated that UnAG blunted the apoptotic alterations including elevations of apoptotic DNA fragmentation index and number of TUNEL-positive nuclei and the upregulation of Bax in the muscle exposed to doxorubicin (Yu et al., 2014). The tumor suppressor protein p53 is known to be involved in DNA damage signaling and is a SIRT1 molecular target (Motta et al., 2004). The notion that p53 contributes to the modulation of apoptotic signaling in ischemia-reperfusion injury (Hatoko et al., 2002) further stimulated our interest in examining its signaling events in the present muscle compression injury study. As a molecular sensor of cellular stress, p53 is activated and phosphorylated on serine-15 (Canman et al., 1998) and then executes Bax-associated apoptotic signal transduction (Vermeulen et al., 2003). In this study, we demonstrated that the contents of total p53, phospho-p53, and p53 phosphorylation ratio were significantly elevated in the compressed muscle in saline control and SIRT1-inhibitor groups, but not in muscle treated only with UnAG. The increase in apoptotic DNA fragmentation index and simultaneous elevation of pro-apoptotic protein Bax in response to p53 phosphorylation is consistent with previous reports (Shieh et al., 1997; Siu et al., 2009; Sin et al., 2013). Our finding that p53 expression was blunted in muscle treated with UnAG, but was significantly elevated in the compressed muscle relative to the uncompressed muscle in saline and SIRT1-inhibitor groups, suggests that SIRT1 was probably involved in mediating the protective effect of UnAG. These results are consistent with the findings of Pinton et al. (2007) who demonstrated that UnAG-induced SIRT1 expression resulted in p53 deacetylation and protection of endothelial cells against senescence in ischemic conditions. The decrease in SIRT1 deacetylase activity in the saline and SIRT1-inhibitor-treated muscles after moderate compression might have resulted from the complicated process of ischemia-reperfusion injury including reactive oxygen species, calcium overload, endothelial dysfunction, inflammatory response (Yellon and Hausenloy, 2007), and regulation via microRNAs (Revollo and Li, 2013). These findings indicated that the possible deacetylation of p53 by SIRT1, in response to UnAG, might provide clues for future investigation of the underlying mechanisms of pressure-induced tissue injury and pressure ulcers. Apoptosis inducing factor (AIF) is a mitochondrial flavoprotein which, upon release, is capable of causing chromatin condensation and DNA fragmentation in the nucleus (Ye et al., 2002). Modjtahedi et al. (2006) observed that the contribution of AIF to cell death induction depended probably on cell type and death triggers. We reported that, in response to compression injury, AIF was concomitantly elevated with the increased Bax expression, except in muscle treated with UnAG. Multi-domain pro-apoptotic proteins Bax and Bak play essential roles in mitochondrial outer membrane permeability, leading to release of AIF in response to cell death trigger (Letai et al., 2002). In this study, we speculate that UnAG might have partly blunted AIF by inhibiting the upstream regulators of apoptosis including p53 and Bax as well as stimulating SIRT1 enzymatic activity. Further study is warranted to examine this speculation and to reveal the exact interaction of UnAG with the p53-Bax-AIF apoptotic signaling axis.

While the present study has supplied useful information about the anti-necroptotic and anti-apoptotic effects of UnAG on muscle compression, which probably involved the mediation of SIRT1 signaling pathway, it has several limitations that need be acknowledged. First, the sole effect of EX527 on compression-induced injury was not investigated in the current study. Besides, data for SIRT1 activity at different time points after EX527 administration might be useful for explaining the unaltered SIRT1 activity of control muscle tissues, as muscle tissues were currently sampled 24 h after EX527 administration. Finally, the present study quantified only the protein levels of RIP1 and RIP3. It is worthwhile to unravel the extent of necroptosis in compression injury by determining the phosphorylation status of necroptosis proteins, especially phospho-Ser161-RIP1.

In conclusion, our data demonstrated that UnAG preserved muscle histology and blunted the alterations of necroptosis and apoptosis signaling in response to compression injury. The observed protective effects were probably associated with the SIRT1 signaling as the effects of UnAG were mostly abolished in the presence of the potent pharmacological inhibitor of SIRT1. Acute cell loss might result from excessive cell death due to ischemia-reperfusion injury (Lorenzo and Susin, 2007), which is a hallmark of pressure-induced tissue injury and pressure ulcers. The present study provided novel data contributing to the understanding of necroptosis and apoptosis on the myoprotective effect of UnAG on pressure ulcers. As UnAG preserved muscle architecture by blunting the compression-induced necroptosis and apoptosis, it might be worthwhile to further explore UnAG as one of the targets to develop new therapeutic regimens.

## Author contributions

FU, AY, and PS: designed the studies; FU, AY, TS, and BT: performed the experiments; FU, AY, TS, BT, and PS: analyzed and interpreted data; FU, CL SW, and PS: contributed to discussion and editing; FU, AY, and PS: supervised the project and co-wrote the manuscript.

### Conflict of interest statement

The authors declare that the research was conducted in the absence of any commercial or financial relationships that could be construed as a potential conflict of interest.
